# Progesterone receptor isoform-dependent cross-talk between prolactin and fatty acid synthase in breast cancer

**DOI:** 10.18632/aging.202289

**Published:** 2020-12-10

**Authors:** Javier A. Menendez, Susan K. Peirce, Adriana Papadimitropoulou, Elisabet Cuyàs, Travis Vander Steen, Sara Verdura, Luciano Vellon, Wen Y. Chen, Ruth Lupu

**Affiliations:** 1 Program Against Cancer Therapeutic Resistance (ProCURE), Metabolism and Cancer Group, Catalan Institute of Oncology, Girona, Spain; 2 Girona Biomedical Research Institute (IDIBGI), Girona, Spain; 3 Peirce Medical Communications, Clemson, SC 29634, USA; 4 Center of Basic Research, Biomedical Research Foundation of the Academy of Athens, Athens, Greece; 5 Mayo Clinic, Division of Experimental Pathology, Department of Laboratory Medicine and Pathology, Rochester, MN 55905, USA; 6 Stem Cells Laboratory, Institute of Biology and Experimental Medicine (IBYME-CONICET), Buenos Aires, Argentina; 7 Department of Biological Sciences, Clemson University, Greenville, SC 29634, USA; 8 Mayo Clinic Minnesota, Department of Biochemistry and Molecular Biology Laboratory, Rochester, MN 55905, USA; 9 Mayo Clinic Cancer Center, Rochester, MN 55905, USA

**Keywords:** luminal breast cancer, prolactin receptor, G129R, endocrine therapy

## Abstract

Progesterone receptor (PR) isoforms can drive unique phenotypes in luminal breast cancer (BC). Here, we hypothesized that PR-B and PR-A isoforms differentially modify the cross-talk between prolactin and fatty acid synthase (FASN) in BC. We profiled the responsiveness of the FASN gene promoter to prolactin in T47D_co_ BC cells constitutively expressing PR-A and PR-B, in the PR-null variant T47D-Y cell line, and in PR-null T47D-Y cells engineered to stably re-express PR-A (T47D-YA) or PR-B (T47D-YB). The capacity of prolactin to up-regulate FASN gene promoter activity in T47D_co_ cells was lost in T47D-Y and TD47-YA cells. Constitutively up-regulated FASN gene expression in T47-YB cells and its further stimulation by prolactin were both suppressed by the prolactin receptor antagonist hPRL-G129R. The ability of the FASN inhibitor C75 to decrease prolactin secretion was more conspicuous in T47-YB cells. In T47D-Y cells, which secreted notably less prolactin and downregulated prolactin receptor expression relative to T47D_co_ cells, FASN blockade resulted in an augmented secretion of prolactin and up-regulation of prolactin receptor expression. Our data reveal unforeseen PR-B isoform-specific regulatory actions in the cross-talk between prolactin and FASN signaling in BC. These findings might provide new PR-B/FASN-centered predictive and therapeutic modalities in luminal intrinsic BC subtypes.

## INTRODUCTION

Prolactin is a well-characterized hormone required for terminal differentiation of mammary epithelial cells and for synthesis of milk components during lactation [1–3]. Beyond its recognized role in the development and differentiation of the normal breast, prolactin causally contributes to the pathogenesis of breast cancer *via* an autocrine/paracrine loop involving prolactin binding to its membrane-associated prolactin receptor (PRLR). Similar to what has been reported for estrogen and progesterone, studies *in vitro* implicate a role for prolactin in breast cancer cell proliferation and survival [4–11], and high levels of this hormone have been shown to drive mammary tumor development in mice [12, 13]. In women, elevated levels of prolactin correlate with increased breast cancer risk and metastasis, whereas lower levels of prolactin/PRLR in clinical samples associate with improved patient survival [14–19]. Early studies with the PRLR antagonist hPRL-G129R – a variant of normal human prolactin with a single amino acid substitution mutation – revealed its capacity to inhibit the prolactin-induced oncogenic signaling responsible for cancer cell proliferation [20–24]. More recently, prolonged treatment with hPRL-G129R in ovarian cancer models was found to antagonize the signaling activities of the prolactin/PRLR tumoral axis and to inhibit tumor growth by inducing destructive autophagy [25].

Despite the biological and clinical relevance of the prolactin/PRLR axis, incomplete knowledge of the underlying network has largely precluded its therapeutic exploitation in specific breast cancer subtypes. Upon engagement of prolactin with PRLR, the resulting activation of JAK/STAT, PI3K, and MAPK signaling pathways enhances the survival, proliferation, differentiation, and motility of normal breast epithelial cells [7]. Activation of these transduction cascades enables not only the expansion of the breast epithelial cell population during pregnancy, but also the differentiation of those epithelial cells responsible for the synthesis and secretion of milk during lactation [7, 26, 27]. This association might similarly lead to augmented growth and motility of breast cancer cells. Although less clearly defined, a loss of responsiveness of breast cancer tissues to the pro-differentiation activities of prolactin might be linked to its pathogenic role in certain breast cancer subtypes and/or disease stages. In this regard, it is well known that prolactin-driven differentiation is characterized by its capacity to orchestrate the expression of key lipid biosynthesis genes and regulate the activity of lipogenic enzymes, leading to cytoplasmic lipid droplets in lactating mammary epithelial cells [28]. A key lipogenic enzyme for the development, functional competence, and maintenance of the lactating mammary gland is fatty acid synthase (FASN), which participates in the prolactin-promoted generation of large quantities of medium- and long-chain fatty acids and total fatty acid contents in milk [29–32]. FASN is a well-characterized driver of metabolic reprogramming in cancer cells [33–35]. Interestingly, the metabolo-oncogenic nature of FASN in breast cancer does not rely on its lactogenic activity, but rather on its ability to provide energy, macromolecules for membrane synthesis, and lipid signals, that facilitate cancer cell survival and proliferation, and also regulate the activity of other oncogenic pathways [33–37]. However, little is known about how prolactin and FASN signaling interact during breast cancer progression. The finding that suppression of FASN-driven endogenous lipogenesis is sufficient to restore normal ductal-like structures in the mammary gland irrespective of the mutational background of undifferentiated malignant phenotypes [37], underpins the notion that FASN gene expression must be closely controlled and regulated for the differentiation and maintenance of normal-like tissue architectures in the breast [38, 39].

The phenotypic effects of prolactin on normal mammary epithelium involve spatio-temporal crosstalk between PRLR and progesterone/progesterone receptor (PR) signaling. Progesterone induces the expression of the PRLR, PR and PRLR cooperate during ductal branch growth in the mammary gland, and PR signaling represses the PRLR-triggered lactogenic signaling that induces milk protein expression [40–42]. Progesterone signaling in breast tissues is mediated by two co-expressed PR isoforms – full-length PR-B and N-terminal truncated PR-A – which regulate the same, as well as distinct, gene sets [43–45]. For instance, whereas PR-A is both necessary and sufficient to elicit the progesterone-dependent reproductive responses for uterine development and fertility, PR-B is required for the normal proliferative and differentiative responses of the mammary gland to progesterone [46–49]. An ever-growing body of complex and sometimes conflicting evidence has shown that isoform-specific PR expression is a context-dependent driver of distinct luminal breast cancer phenotypes in terms of the endocrine sensitivity, proliferative capacity, and cancer stem-like cell behavior [50–62]. However, how the close functioning of the progesterone/PR and prolactin/PRLR signaling axes that drive the lactogenic/lipogenic phenotypic outcomes in normal mammary gland [63] might be altered in breast cancer tissues remains largely unexplored. Here, we tested the hypothesis that the PR-B and PR-A isoforms differentially modify the ability of prolactin to transcriptionally regulate the expression of the FASN gene in PR+ breast cancer cells. We also tested the contrary hypothesis that prolactin secretion and/or PRLR expression are affected by pharmacological interruption of FASN signaling.

## RESULTS

### Expression of FASN and PRLR mRNAs is significantly elevated in PR-positive breast cancer

**Figure 1:**
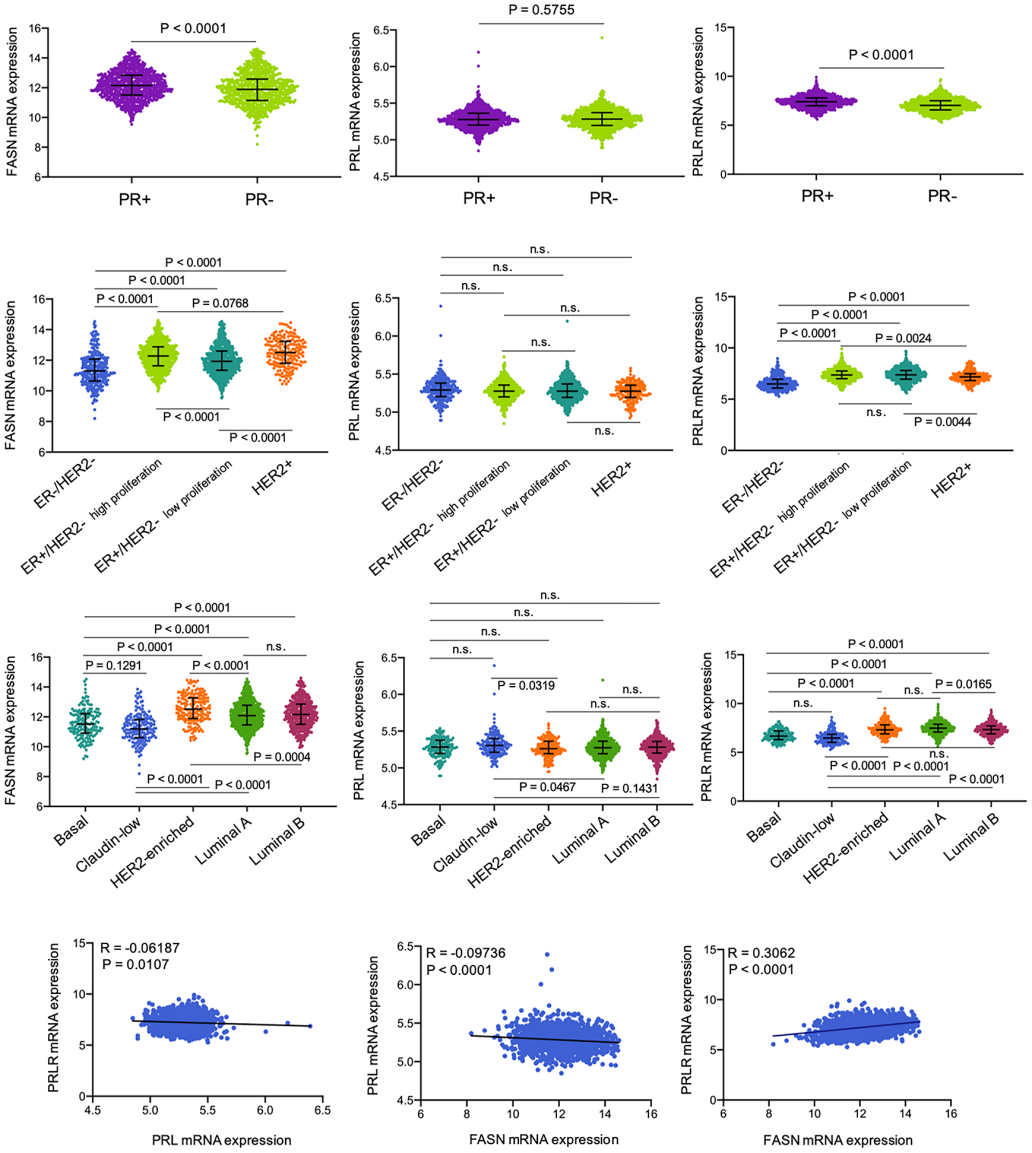
**Differential enrichment of FASN, prolactin, and PRLR genes in breast cancer subtypes.** FASN, prolactin, and PRLR mRNA expression levels in primary breast tumors from the METABRIC project classified into distinct subtypes using different classifiers. (PR+, n=903 *versus* PR-, n=797, Mann Whitney test; 3-genes signature, estrogen receptor (ER)-/HER2-, n=290, ER+/HER2- high proliferation, n=603, ER+/HER2+ low proliferation, n=619, HER2+, n=188, ANOVA with Dunn’s multiple comparison test; PAM50: basal, n=161, claudin-low, n=186, HER2 enriched, n=190, luminal A, n=631, luminal B, n= 412, ANOVA with Dunn’s multiple comparison test).

FASN is an endogenous PR-responsive gene [64–67], which might explain, at least in part, the simultaneous increase in expression of FASN and PR proteins early during human mammary carcinogenesis [68]. We interrogated transcriptional data from the METABRIC [69, 70] to explore the association between FASN and PR in breast cancer (Figure 1). FASN mRNA expression was significantly higher in PR-positive breast tumors than in PR-negative tumors (p < 0.0001; n=1,700), as determined by PR gene expression profiles. PRLR expression was also significantly elevated in PR-positive tumors (p < 0.0001), whereas prolactin expression was not significantly different between the two subgroups (p = 0.5755). It was therefore of interest to evaluate whether FASN mRNA expression correlated with that of PRLR. PRLR was among the top-100 genes positively correlated with FASN gene expression (r = 0.31, p < 0.0001) in the METABRIC breast cancer dataset. Indeed, when the data set was classified according to the five intrinsic subtypes (luminal-A, luminal-B, HER2-enriched, claudin-low, and basal-like) using the research-based 50-gene prediction analysis of microarray (PAM50) classifier [71], both FASN and PRLR (but not prolactin) mRNAs were found to be significantly higher in HER2-enriched and luminal subtypes than in highly aggressive/poor prognosis basal-like and claudin-low subtypes (Figure 1). The highest levels of FASN and PRLR mRNAs were detected in HER2-enriched and luminal-A subtypes, respectively.

### Maximum prolactin secretion and PRLR expression requires both PR-A and PR-B isoforms

**Figure 2:**
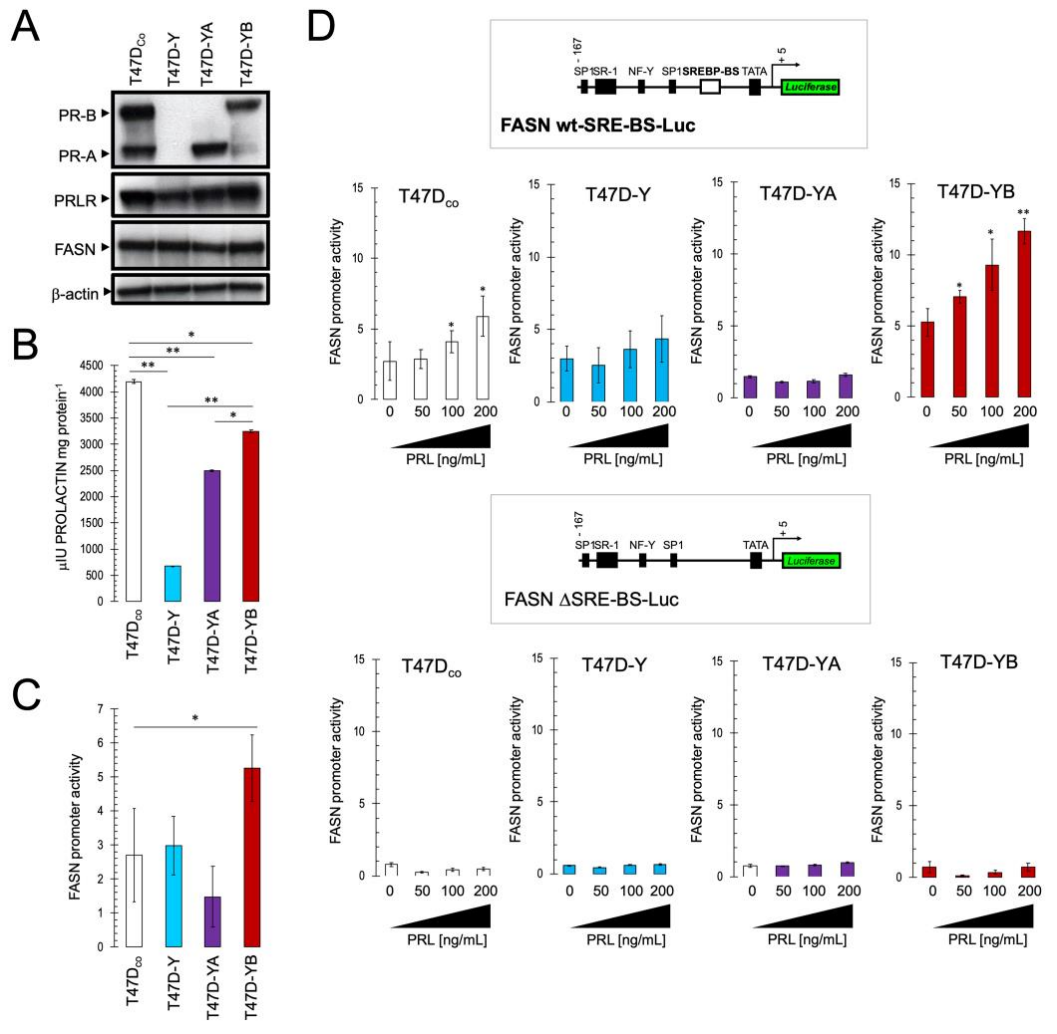
**Exogenous prolactin activates the FASN gene promoter in a PR-B/SBREP-dependent manner.** (**A**). Immunoblotting of baseline expression status of PR-A, PR-B, PRLR, and FASN proteins in T47D_co_, T47D-Y, T47D-YA and T47D-YB breast cancer cell lines. β-actin was used to control for protein loading and transfer. (**B**). Immunoassay-based quantification of baseline autocrine prolactin secretion into the extracellular milieu of T47D_co_, T47D-Y, T47D-YA and T47D-YB breast cancer cell lines. (**C**, **D**). Estradiol-depleted cells were transiently transfected with a plasmid containing a luciferase gene driven by a 178-bp FASN gene promoter fragment harboring a SREBP-binding site, flanked by auxiliary NF-Y and Sp-1 sites or with a similar construct in which the SREBP domain was deleted. The next day, cells were treated with graded concentrations of recombinant prolactin (PRL) in 0.5% CCS. After 24 h, cells were lysed and luciferase activity was measured. Luciferase activity was expressed as relative (fold) change in transcriptional activities of FASN promoter-transfected cells in response to prolactin treatment after normalization to pRL-CMV activity. Each experimental value represents the mean fold increase (columns) ± S.D. (bars) from at least three separate experiments in which triplicate wells were measured. Luciferase activity in prolactin-treated cells was compared with that in vehicle-treated control cells (* P < 0.05; ** P < 0.005).

To evaluate the relevance of PR isoform expression and ratio on the regulatory activity of prolactin for FASN gene expression, we used the PR-A/PR-B-positive (T47D_co_) and PR-null (T47D-Y) variants of the estrogen receptor (ER)/PR-positive breast cancer cell line T47D. T47D_co_ cells endogenously express equimolar levels of PR-A and PR-B in a constitutive and estrogen-independent manner, thereby allowing the study of the functional relevance of PR without the confounding effects of estrogen [44, 62, 72–75]. T47D-Y cells can also be used to determine the effect of PR isoform variants or mutants by stably reintroducing PR-A or PR-B on expression vectors. The resulting cell lines, termed T47D-YA (stably expressing the full-length PR-A isoform) and T47D-YB (stably expressing the full-length PR-B isoform), have been widely employed to evaluate the independent signaling function of each PR isoform [43, 62, 72–75]. Here, we employed all four T47D cell lines, each with a different PR content (Figure 2A), to test for isoform-specific prolactin gene regulation and functional interactions with the FASN gene.

ELISA-based quantification of prolactin content in the conditioned medium of T47D_co_, T47D-Y, T47D-YA, and T47D-YB cultures revealed considerably lower amounts (~85%) in PR-null T47D-Y cultures than in T47D_co_ cultures (Figure 2B). Although the individual re-expression of each PR isoform was not sufficient to reach the levels of prolactin found in T47D_co_ cultures, the stable re-expression of PR-A and PR-B notably augmented by 3.7- and 4.8-fold, respectively, the extracellular amounts of prolactin in T47D-Y cultures. We also compared the four cell types in terms of PRLR abundance by immunoblotting equivalent cell extracts using an antibody against PRL4 (Figure 2A). Analysis showed that T47D_co_ cells likewise harbored an abundant amount of PRLR, whereas a notably decreased amount of PRLR was detected in PR-null T47D-Y cells. T47D-YB cells expressed PRLR at slightly higher levels than T47D-YA cells. These findings, altogether, reveal not only a close correlation between the status of prolactin secretion and PRLR expression, but also that both PR-A and PR-B are required to achieve maximum levels of prolactin secretion and PRLR expression.

### Progesterone receptor isoforms differentially impact baseline FASN expression

To assess how the PR status might alter baseline FASN promoter activity, T47D_co_, T47DY, T47D-YA, and T47D-YB cells were transfected with a luciferase reporter vector encoding a sterol regulatory element-containing FASN promoter sequence fused with firefly luciferase. As shown in Figure 2C, the absence of both PR isoforms failed to alter luciferase expression in T47D-Y cells with respect to baseline levels in T47D_co_ cells. Intriguingly, FASN promoter activity was reduced by approximately 50% in cells exclusively expressing the PR-A isoform (T47D-YA), but increased by more than 90% in cells exclusively expressing the PR-B isoform (T47D-YB). At the protein level, immunoblotting confirmed the lower amounts of FASN in T47D-YA cells and the slightly higher FASN levels in T47D-YB cells (Figure 2A).

### Prolactin-induced up-regulation of FASN gene expression is PR-B isoform-specific

Transient transfection experiments with the FASN reporter demonstrated the ability of graded concentrations of prolactin (50, 100, and 200 ng/mL) to dose-dependently up-regulate (up to 2.1-fold at 200 ng/mL prolactin) promoter activity in T47D_co_ cells (Figure 2D, top). PR-null T47D-Y cells, however, remained mostly insensitive to the regulatory effects of prolactin on the FASN reporter. We examined PR isoform-dependent, prolactin-driven FASN promoter activity, finding that T47D cells containing PR-A (T47D-YA cells) were completely unresponsive to prolactin. By contrast, the sole presence of PR-B preserved the ability of prolactin to increase FASN promoter activity, as previously observed in T47D_co_ cells (Figure 2D, top). Indeed, the presence of PR-B in the absence of PR-A significantly boosted the response of the FASN promoter to prolactin concentrations as low as 50 ng/mL, suggesting that prolactin-induced activation of the FASN reporter is largely mediated through PR-B. We further found that prolactin-induced FASN promoter activity was blocked in cells transiently transfected with a truncated version of the proximal FASN gene promoter in which the region responsible for SREBP binding was deleted (FASNΔSRE; Figure 2D, bottom), overall suggesting that prolactin activates the FASN promoter primarily through the SREBP1 regulatory site [76–78].

### The PRLR-specific antagonist hPRL-G129R impedes prolactin-driven activation of FASN gene expression

**Figure 3:**
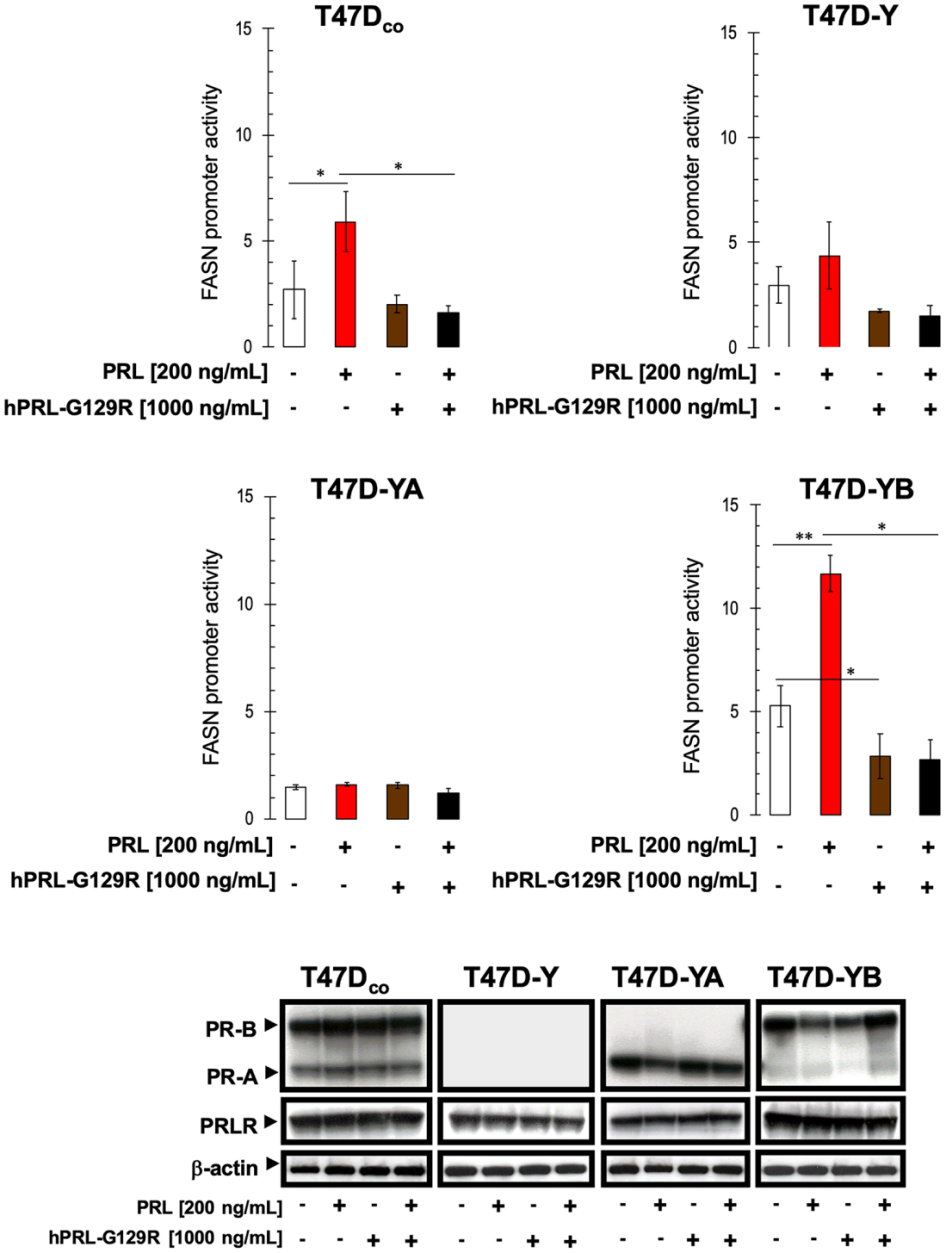
**Exogenous prolactin activates FASN gene promoter activity by engaging PRLR.** *Top.* Estradiol-depleted cells were transiently transfected with a plasmid containing a luciferase gene driven by a 178-bp FASN gene promoter fragment harboring a SREBP-binding site, flanked by auxiliary NF-Y and Sp-1 sites as described in Figure 2C, 2D. The next day, cells were treated with 200 ng/mL prolactin (PRL) in the absence or presence of a 5-fold-excess of the prolactin antagonist hPRL-G129R (1000 ng/mL) in 0.5% CCS. After ~24 h of incubation, cells were lysed, luciferase activity was measured and relative (fold) changes in transcriptional activities of FASN promoter-luciferase-transfected cells were calculated. The data are shown as the means (*columns*) ± S.D. (*bars*) from three separate experiments (performed in duplicate). Luciferase activity in prolactin- and/or hPRL-G129R-treated cells was compared with that in vehicle-treated control cells (* P < 0.05; ** P < 0.005). *Bottom*. Estradiol-depleted cells were treated with 200 ng/mL PRL in the absence or presence of a 5-fold excess of hPRL-G129R in 0.5% CCS for 48 h. Immunoreactive bands for PR-A, PR-B, and PRLR proteins were analyzed by immunoblotting as described in Figure 2A. β-actin was used to control for protein loading and transfer. Figure shows a representative immunoblot analysis. Similar results were obtained in 3 independent experiments.

To substantiate that prolactin activates FASN gene expression by its engagement with PRLR, we used the PRLR-specific antagonist hPRL-G129R [20–25, 79–82]. Prolactin-induced activation of FASN gene expression (at 200 ng/mL) in T47D_co_ and T47D-YB cells was completely inhibited by co-incubation with hPRL-G129R at a relatively low concentration of 1000 ng/mL (i.e., 5-fold-excess of prolactin) (Figure 3, top). Further, PRLP-specific blockade with hPRL-G129R sufficed to return the overactive FASN promoter in T47D-YB cells to the baseline state seen in T47D_co_ cells. Immunoblotting assays failed to demonstrate any significant changes in PRLR expression in response to prolactin, hPRL-G129R, or their combination (Figure 3, bottom); however, hPRL-G129R co-treatment prevented the ability of prolactin to marginally down-regulate PR-A and PR-B expression in T47D-YA and T47D-YB cells, respectively. These findings, altogether, imply that the prolactin-FASN signaling axis acts through PRLR in T47D cells, and suggest the specific functional engagement of the PR-B isoform as the driver of FASN gene responsiveness to the regulatory effects of prolactin/PRLR.

### FASN inhibition suppresses PR-A and PR-B expression

We previously demonstrated that FASN blockade suppresses the well-documented capacity of estradiol to up-regulate PR expression in endometrial cancer cells [83, 84]. We therefore explored the possibility that FASN signaling regulates the constitutive expression of both PR isoforms irrespective of estradiol stimulation. As shown in Supplementary Figure 1**,** the expression of PR-A and PR-B in T47D_co_ cells was dose-dependently suppressed in the presence of graded concentrations of the FASN inhibitor C75. Indeed, PR-A and PR-B expression was very low to undetectable in the presence of 10 μg/mL C75 not only in T47D_co_ cells but also in T47D-YA and T47D-YB cells (Supplementary Figure 1).

### FASN inhibition modifies prolactin secretion and PRLR expression in a PR isoform-dependent manner

**Figure 4:**
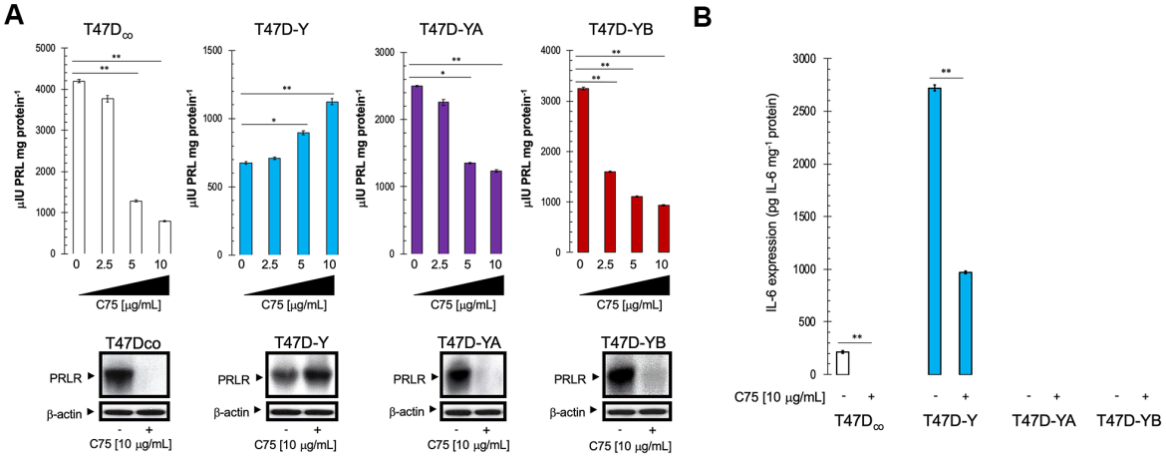
**Pharmacological blockade of FASN activity modifies autocrine prolactin secretion in a PR-dependent manner.** (**A**). *Top.* Autocrine prolactin secretion levels in the extracellular milieu of estradiol-depleted cells cultured in the absence or presence of graded concentrations of C75 in 0.5% CCS for 48 h were determined by a commercially available EASIA kit. Data are means (*columns*) ± S.D. (*bars*) from three independent experiments performed in duplicate. Secreted amounts of prolactin in C75-treated cells were compared with those in vehicle-treated control cells (* P < 0.05; ** P < 0.005). *Bottom.* Cell lysates strictly obtained from the same experimental replicates employed in A were subjected to immunoblotting for PRLR protein expression. β-actin was used to control for protein loading and transfer. Figure shows a representative immunoblot analysis. Similar results were obtained in 3 independent experiments. (**B**). Autocrine IL-6 levels in the extracellular milieu of estradiol-depleted cells cultured in the absence or presence of 10 μg/mL C75 in 0.5% CCS for 48 h were determined by a commercially available ELISA kit. Secreted amounts of IL-6 in C75-treated cells were compared with those in vehicle-treated control cells (** P < 0.005).

We next tested the hypothesis that FASN signaling regulates prolactin secretion in a PR isoform-dependent manner (Figure 4A). Prolactin secretion in T47D_co_ cells was reduced by C75 in a dose-dependent manner (up to approximately 80% suppression at 10 μg/mL C75; Figure 4A, top). In T47D-YA cells, however, prolactin secretion was significantly reduced by only 50% at the same concentration of C75. Conversely, C75 concentrations as low as 2.5 μg/mL sufficed to decrease prolactin secretion by 50% in T47-YB cells, whereas the suppression of secretion as high as 80% was observed at 10 μg/mL C75 (Figure 4A, top). A significant, dose-dependent up-regulation of prolactin secretion occurred in PR-null T47D-Y cells exposed to graded concentrations of C75 (up to approximately 170%). We then explored the ability of FASN signaling to regulate PRLR expression. Remarkably, we observed the complete loss of PRLR expression in T47D_co_, TD47Y-A, and T47D-YB cells grown in the presence of C75 (Figure 4A, bottom). By contrast, PRLR protein expression in PR-null Y47D-Y cells was conspicuously up-regulated in response to C75 (Figure 4A, bottom.

### FASN inhibition suppresses epithelial-to-mesenchymal transition-related aggressiveness in PR-null T47DY cells

Both PR and PRLR have been suggested to act as as promoters of more differentiated phenotypes *via* suppression of the epithelial-to-mesenchymal (EMT) program. Indeed, loss of PR in T47D-Y cells has been associated with a change in cell morphology to a more mesenchymal-like phenotype, accompanied by increased cell motility, and up-regulation of EMT-associated genes [85, 86]. Similarly, prolactin blockade in epithelial-like breast cancer cells has been shown to induce mesenchymal-like phenotypic changes and enhance invasiveness, whereas activation of PRLR in mesenchymal-like breast cancer cells suppresses mesenchymal properties and reduces invasive behaviors [87, 88]. A robust surrogate marker of aggressive breast cancer phenotype *via* regulation of EMT is interleukin-6 (IL-6) [89–92]. We therefore investigated whether the apparent ability of FASN blockade to restore a prolactin autocrine function in T47D-Y cells *via* augmented secretion of PR and re-activation of PRLR expression might impact the EMT-related status of IL-6 expression. EMT-like PR-null T47D-Y cells released extremely high levels of IL-6 into the extracellular milieu when compared with PR-A/-B-expressing T47D_co_ parental cells (approx. 13-fold increase; Figure 4B). The FASN inhibitor C75 not only suppressed the baseline IL-6 expression in T47D_co_ parental cells, but further reduced (by more 50%) the augmentation of IL-6 secretion promoted by loss of PR in T47D-Y cells (Figure 4B).

### PRLR inhibition decreases FASN expression in HER2-overexpressing breast cancer cells

**Figure 5:**
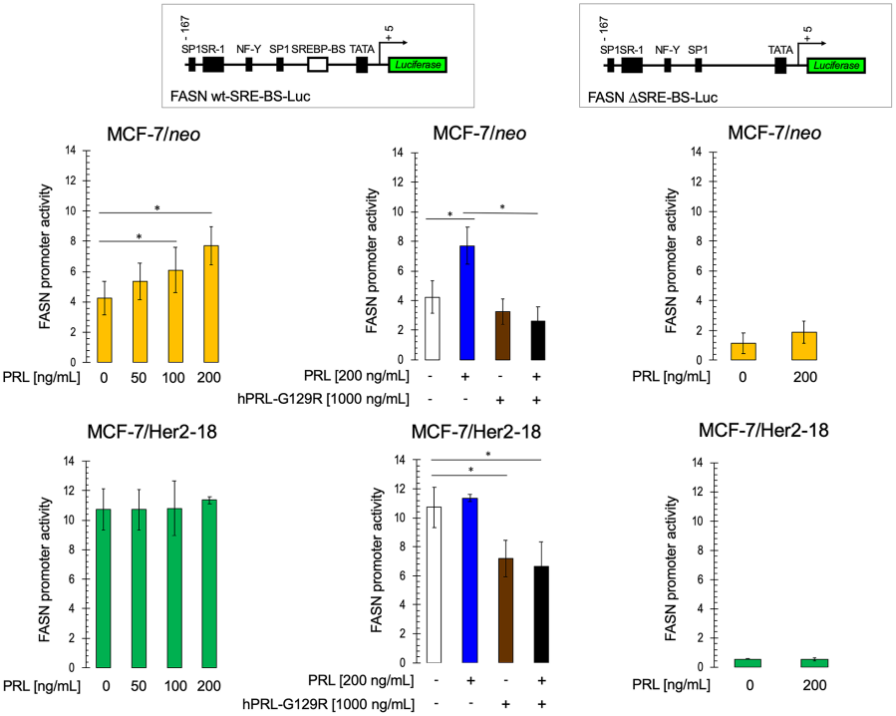
**HER2 overexpression prevents prolactin-induced activation of the FASN gene promoter.** Estradiol-depleted MCF-7/neo and MCF-7/Her2-18 cells were transiently transfected with a plasmid containing a luciferase gene driven by a 178-bp FASN gene promoter fragment harboring a SREBP-binding site, flanked by auxiliary NF-Y and Sp-1 sites or with a similar construct in which the SREBP domain was deleted. The next day, cells were treated with recombinant prolactin (PRL) in the presence or absence of hPRL-G129R in 0.5% CCS. After ~24 h of incubation, cells were lysed, luciferase activity was measured and relative (fold) changes in transcriptional activities of FASN promoter-luciferase-transfected cells were calculated. The data are shown as the means (*columns*) ± S.D. (*bars*) from three separate experiments (performed in duplicate). Luciferase activity in PRL- and/or G129R-treated cells was compared with that in vehicle-treated control cells (* P < 0.05; ** P < 0.005).

Several studies suggest that there is potential cooperation between PRLR and HER2 during breast cancer progression [82, 93, 94]. We speculated that, if a cross-talk between HER2 and autocrine prolactin/PRLR signaling is actively involved in the well-known FASN overexpressing-phenotype of HER2-positive breast cancer cells [36, 95–98], blockade of PRLR should then reduce the ability of HER2 to constitutively up-regulate FASN gene expression. We found that whereas exogenous stimulation with prolactin dose-dependently increased FASN promoter activity in HER2-negative MCF-7/neo control cells, it failed to promote any further increase in activity of the already hyperactive FASN gene promoter in MCF-7/HER2 cells (Figure 5). hPRL-G129R fully prevented the positive regulatory effects of prolactin on the FASN gene in MCF-7/neo parental cells and partially reduced the overactive FASN gene promoter in MCF-7/HER2 cells, thus suggesting that PRLR signaling actively engages HER2 signaling to fully hyperactivate the FASN gene promoter in these cells (Figure 5).

## DISCUSSION

Prolactin has a central role in mammary gland development and terminal differentiation of mammary epithelial cells. The prolactin/PRLR-signaling axis has been consistently shown to play a permissive role in the development of primary breast carcinomas and distant metastatic lesions [3, 5, 7, 12, 13, 18, 20, 22, 99, 100]. Recent studies have, however, questioned the role of prolactin in breast cancer development/progression and have highlighted a putative suppressor role in breast tumorigenesis [87, 101]. This latter role is supported by the association between prolactin/PRLR down-regulation and significantly better survival outcome in patients with breast cancer [88, 102–105], which is consistent with the lack of anti-tumorigenic effects and therapeutic benefits observed in clinical trials with PRLR antagonists [106]. Accordingly, the restoration/activation of prolactin/PRLR signaling has been shown to promote cell differentiation and reverse highly proliferative, invasive, mesenchymal and tumorigenic phenotypes, such as those of the ER/PR double-negative MDA-MB-231 breast cancer model [107]. How might we reconcile these apparently conflicting pro- and anti-tumorigenic roles of prolactin?

We took advantage of the T47D_co_ luminal breast cancer cell line, which constitutively express high levels of ER and PR, and allows the study of the unliganded, progesterone-independent regulatory effects of human PRs in ER-positive luminal-like breast cancer without the confounding requirement of estradiol to stimulate PR expression. In addition to this, we employed the PR-negative T47D subline T47D-Y, in which cloning approaches restored either PR-A or PR-B expression, and used these cells to study the regulatory roles of each isoform in isolation [72, 108]. Because all of these models share the natural background of PR+ luminal breast cancer, in which we detected a positive correlation between PRLR and FASN genes in the METABRIC repository, they are expected to contain the appropriate ancillary coregulatory factors needed for faithful regulation of prolactin/PR-dependent genes. Our results lead us to propose that the PR isoform-specific regulation of distinct regulatory responses in the cross-talk between prolactin and FASN might be involved in the diverse phenotypic outcomes arising from the prolactin/PRLR signaling axis in luminal breast cancer.

**Figure 6:**
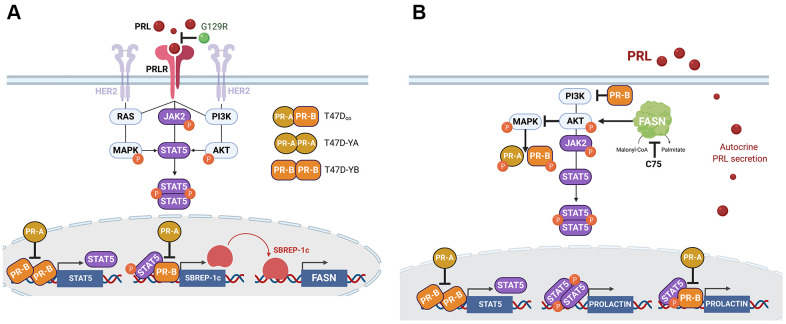
**A PR isoform-dependent cross-talk between prolactin and FASN in breast cancer: a working model.** The convergence of prolactin/PRLR and PR signaling interactions on STAT5 [130, 131] might explain, at least in part, the PR-B-driven capacity of prolactin to activate FASN in luminal breast cancer cells (**A**). Similar inputs, likely involving yet-to-be explored phospho-modifications of PR isoforms, might underlie also the ability of FASN signaling to regulate autocrine prolactin secretion (**B**).

We first assessed how PR-B and PR-A isoforms could modify the ability of prolactin to regulate the expression of the FASN gene in PR+ breast cancer cells. Historically, PR-B has been characterized as a strong “positive” regulator of the effects of progesterone, whereas PR-A is often regarded as a ligand-independent mediator of gene repression. Also, the isoforms differ with regard to the positive *versus* negative regulatory direction and also the type of regulated genes. Thus, whereas PR-B mostly regulates the expression of genes required for cell proliferation [43], PR-A mainly controls the expression of genes involved in cell adherence, cell morphology, and resistance to apoptosis [50, 109, 110]. We found that the ability of the prolactin/PRLR signaling to transcriptionally up-regulate FASN gene expression was specifically dictated by: a.) the obligatory presence of PR-B to enable prolactin-driven FASN gene activation, and b.) the necessary lack of PR-A to facilitate maximum FASN gene activation in response to prolactin. Accordingly, whereas PR-B+ T47-YB cells were exquisitely responsive to exogenous prolactin in terms of FASN gene up-regulation, both PR-null T47D-Y and PR-A+ T47D-YA cells remained completely unresponsive to the FASN regulatory effects of prolactin. When both PR isoforms are co-expressed (i.e., T47D_co_), prolactin-induced FASN activation is considerably dampened relative to that of cells exclusively expressing PR-B (i.e., T47D-YB). PR-A trans-repression of PR-B might explain this observation and likely implicates a negative effect of PR-A within PR heterodimers, as has been reported in multiple models [110–115]. In this case, however, how do PR isoform-specific signaling events impact the ability of the same “classical” prolactin/PRLR/JAK2/STAT5 pathway to differentially mediate prolactin-induced FASN gene transcription? Of note, the ability of PR-B to facilitate prolactin-driven FASN activation required an SREBP-binding site in the FASN gene promoter. Therefore, PR isoform-specific actions might not simply be explained in terms of STAT5-driven transcriptional regulation of the FASN gene promoter, but instead suggest that the distinct ability of each PR isoform to cooperate with STAT5 would differentially impact SREBP-1c expression. The prolactin/PRLR/JAK2/STAT5 signaling pathway drives fat synthesis and proliferation *via* augmented expression of SREBP-1c [116, 117]. STAT5 modulates the expression and nuclear distribution of SREBP1, thereby regulating its biological functions including the regulation of lipogenic genes such as FASN. In fact, downregulation of (phospho-active) STAT5 decreases, whereas its overexpression increases, the activation of the SREBP1 promoter [118]. Intriguingly, the ability of PR to drive JAK/STAT-dependent transcriptional responses requires the so-called CD domain of PR-B, which is located in the N-terminal B-upstream segment (BUS) region of full-length PR-B – and absent in PR-A – and is essential for proliferative signaling in breast cancer cells [119, 120]. The CD domain of PR-B is required for PR-B-dependent expression of STAT5 which, in turn, then complexes with PR-B on a specific subset of PR-target genes. Indeed, STAT5 appears to operate as a pioneer transcription factor that “opens” sites in chromatin for subsequent PR-B-driven transcriptional activation of target genes [120]. It is therefore tempting to speculate that the BUS region of PR-B might function as the shared signaling hub of the prolactin/PRLR-driven STAT5 and PR-B (but not PR-A) signaling axes. By placing the lipogenic master regulator SREBP-1c as one of the select target genes coregulated by STAT5 and PR-B, we can explain the PR-B isoform-specific regulatory actions on the cross-talk between prolactin and FASN signaling in luminal breast cancer cells (Figure 6). In a scenario in which constitutive activation of PRLR and HER2 (e.g., MCF-7/HER2 cells) leads to a dissociation between prolactin/PRLR and inducible STAT5 activation, which becomes constitutive *via* hyperactivation not only of JAK2 but also of PI3K/AKT/mTOR and MAPK transducers, maximal transcriptional activation of the FASN gene is no longer responsive to exogenous stimulus with prolactin, but can be partially prevented by PRLR antagonists such as a hPRL-G129R [82] (Figure 6).

We envisaged that prolactin secretion and/or PRLR expression might be affected by pharmacological blockade of FASN activity. The achievement of higher levels of autocrine prolactin secretion (and PRLR expression) obligatorily required the presence of the PR-B isoform. Thus, PR-A+ T47-YA cells and, more notably, PR-null T47D-Y cells, secreted less prolactin and downregulated PRLR expression in comparison with PR-A+/PR-B+ T47Dco cells. With the sole exception of sexual hormone (estradiol and androgen)-induced transcriptional up-regulation of autocrine prolactin in rat trigeminal neurons, pituitary lactotrophs, and rat prostate [121, 122], the pathway(s) that regulate the synthesis and secretion of autocrine prolactin in breast cancer cells are largely unknown. The PTEN/PI3K-AKT pathway – one of the most commonly activated metabolic drivers of cancer – is an upstream regulator of autocrine prolactin production in the normal mammary gland; moreover, autocrine prolactin production is a direct mechanism by which PI3K-AKT activation results in PRLR/JAK2/STAT5 pathway activation [123]. Importantly, whereas AKT-induced up-regulation of autocrine prolactin does not require intact PRLR/JAK/STAT5 signaling, the PRLR/JAK/STAT5 pathway is required to mediate the effects of AKT on lipid synthesis in the normal mammary gland [123]. We found that the ability of the FASN inhibitor C75 to reduce prolactin secretion in PR-A+/PR-B+ T47Dco parental cells was either exacerbated in PR-B+ T47D-YB cells or partially dampened in PR-A+ T47-YA cells. Because AKT is rapidly repressed in response to C75-induced blockade of FASN activity [124, 125], our findings suggest a positive feedback regulation between FASN and autocrine prolactin expression via AKT-driven activation of the PRLR/JAK/STAT5 pathways in PR+ breast cancer cells. This positive feedback, however, is apparently fine-tuned by PR signaling in a PR isoform-specific manner, which might reflect, at least in part, the ability of PR-B to cause down-regulation of the PI3K/AKT signal via upregulation of PTEN [126]. In T47D-Y cells lacking PRs and exhibiting a more aggressive undifferentiated phenotype, the suppression of FASN signaling was accompanied by a partial recovery of prolactin secretory activation, up-regulation of PRLR expression, and down-regulation of EMT/cancer stem cell/pro-inflammatory markers such as IL-6. These findings, overall, suggest that the ability of FASN signaling to enable secretory activation of endocrine prolactin is a PR-dependent event that might dictate the level of luminal cell differentiation [38]. Further characterization of the FASN-centered relationship between PRs, prolactin/PRLR/JAK/STAT5, and PI3K/AKT pathways will be required to determine whether therapeutic approaches directed at blocking the interaction between these pathways would be more beneficial to therapeutically manage prolactin and/or FASN signaling in breast cancer cells. Nonetheless, it is important to acknowledge that we employed the FASN inhibitor C75 in the form of a racemic mixture of (-) and (+) enantiomers, which differ in their regulation of FASN and carnitine palmitoyltransferase-I (CPT-1) [127–129]. An evaluation of clinical-grade FASN inhibitors devoid of CPT-I inhibitory activity should definitely clarify the mechanistic role of FASN as a therapeutic target for differentiation therapy in certain subsets of ER+/PR- breast carcinomas [38]

In summary, our data reveal an unforeseen PR-B isoform-specific regulatory action on the cross-talk between prolactin and FASN signaling in luminal breast cancer cells. Our data suggest that the lipogenic FASN might be incorporated into the group of metabolic markers specifically enriched by PR-B (but not with those related to the malignant metabolism of cancer stem cells enriched by PR-A) in luminal A-like PR+ breast cancer cells [130–133], likely promoting survival, proliferation, and differentiation. In ER-positive/PR-negative luminal B-like breast cancer cells, however, FASN signaling might be co-opted as a negative regulator of the epithelial cell phenotype and, accordingly, its blockade might promote the restoration and activation of prolactin/PRLR-driven differentiation programs. Nonetheless, because the PR isoform ratio is a proxy for the molecular signature and endocrine therapy responsiveness of PR+ breast cancer cells, our findings might illuminate new PR-B/FASN-centered predictive and therapeutic modalities in luminal breast cancer intrinsic subtypes.

## MATERIALS AND METHODS

### Reagents

Anti-PR (clone hPRa2 + hPRAa3, Ab-8) and anti-PRLR (Ab-1) mouse monoclonal antibodies were purchased from Lab Vision Corporation (Fremont, CA). Anti-FASN mouse monoclonal antibody (clone 23) was purchased from BD PharMingen Laboratories (San Diego, CA). Anti-β-actin goat polyclonal antibody was purchased from Santa Cruz Biotechnology, Inc. (Santa Cruz, CA).

### Profiling of breast cancer datasets

We interrogated the publicly available Molecular Taxonomy of Breast Cancer International Consortium (METABRIC) breast cancer dataset from the UK and Canada, in which mRNA expression was measured using the Illumina HT-12v13 platform and copy number alterations with the Affymetrix SNP 6.0 array. Gene-level expression files from METABRIC were downloaded from the cBioportal for Cancer Genomics (https://www.cbioportal.org/). We used the 3-gene and PAM50 breast cancer intrinsic subtypes provided in the METABRIC dataset.

### Cell lines

Human T47D_co_ breast cancer cells co-expressing PR-A and PR-B, T47DY cells lacking PRs, T47D-YA cells expressing only PR-A, and T47D-YB cells expressing only PR-B were generously provided by Dr. K. B. Horwitz (University of Colorado). MCF-7/neo and MCF-7/Her2-18 breast cancer cells stably overexpressing the HER2 oncogene were kindly provided by Dr Mien-Chie Hung (University of Texas M.D. Anderson Cancer Center). Cells were grown in Improved Minimal Essential Medium (IMEM) with 5% fetal bovine serum and 2 mmol/L L-glutamine. Before starting any experimental treatment, cells were cultured and washed extensively with phenol red-free IMEM supplemented with 5% dextrin-coated charcoal-treated bovine serum (CCS) for 3 days to ensure complete depletion of estradiol-like compounds from the media.

### Immunoblotting

Cells were washed twice with phosphate buffered saline (PBS) and lysed in a lysis buffer (20 mM Tris [pH 7.5], 150 mM NaCl, 1 mM EDTA, 1 mM EGTA, 1% Triton X-100, 2.5 mM sodium pyrophosphate, 1 mM β-glycerolphosphate, 1 mM Na_3_VO_4_, 1 μg/ml leupeptin, 1 mM phenylmethylsulfonylfluoride) for 30 min on ice. Lysates were cleared by centrifugation in an Eppendorf tube (15 min at 14 000 rpm, 4° C). Protein content was determined against a standardized control using the Pierce Protein Assay Kit (Rockford, IL). Equal amounts of protein (50 μg in the case of PR and PRLR, 10 μg in the case of FASN) were resuspended in 5× Laemmli sample buffer for 10 min at 70° C, subjected to electrophoresis on either 10% SDS-PAGE gels (Novex, San Diego, CA) in the case of PRs and PRLR or 3–8% NuPAGE Tris-Acetate gels (Novex) in the case of FASN, and transferred to nitrocellulose membranes. Nonspecific binding was minimized by blocking for 1 h at room temperature with TBS-T (25 mM Tris-HCl, 150 mM NaCl [pH 7.5], and 0.05% Tween 20) containing 5% (*w/v*) nonfat dry milk. Membranes were then washed in TBS-T and incubated for 2 h at room temperature with specific primary antibodies in TBS-T/5% (*w/v*) nonfat dry milk. Membranes were washed again in TBS-T, horseradish peroxidase-conjugated secondary antibodies (Jackson Immunoresearch Labs, West Grove, PA) in TBS-T were added for 1 h, and immunoreactive bands were detected by enhanced chemiluminescence reagent (Pierce). Blots were re-probed with an antibody for β-actin to control for protein loading and transfer. Figures show representative immunoblot analyses. Similar results were obtained in 3 independent experiments.

### FASN gene promoter activity

To analyze FASN gene promoter activity, estradiol-depleted cells seeded in 24-well plates (~ 5 × 10^4^ cells/well) were transfected (FuGENE 6; Roche Biochemicals, Indianapolis, IN) in low-CCS (0.5% CCS) IMEM with 300 ng/well of the pGL3-luciferase (Promega, Madison, WI) construct containing a luciferase reporter gene driven by either an intact 178-bp FAS promoter fragment harboring a well-characterized SREBP-binding site flanked by auxiliary NF-Y and Sp-1 sites, or with a similar construct in which the SREBP-binding site was deleted. Cells were contransfected with 30 ng/well of the internal control plasmid pRL-CMV, which was used to correct for transfection efficiency. After 18 h, the transfected cells were washed and then incubated in 0.5% CCS for approximately 24 h. Luciferase activity was detected using the Luciferase Assay System (Promega) with a TD-20/20 luminometer (Turner Designs, Sunnyvale, CA). The magnitude of activation in FASN promoter-luciferase-transfected cells was determined after normalization to the luciferase activity in cells co-transfected with equivalent amounts of the empty pGL3-luciferase vector (∅-Luc) and the internal control plasmid pRL-CMV, which was taken as 1.0-fold. This control value was used to calculate the relative (fold) change in transcriptional activities of FASN promoter-luciferase-transfected cells in response to treatments after normalization to pRL-CMV activity, and the data are shown as the means (columns) ± S.D. (bars) from three separate experiments (performed in triplicate).

### Prolactin and IL-6 secretion

T47D_co_, T47D-Y, T47-YA and T47-YB cells were depleted of estradiol by treatment with 5% CCS for 3 days, washed twice with pre-warmed PBS and cultured in serum-free medium overnight. Cells were then cultured in 0.5% CCS for up to 48 h in the absence or presence of graded concentrations of the synthetic FASN inhibitor C75. After this, the conditioned medium was collected, centrifugated at 1,000 × *g* for 10 min at 4° C to remove debris, and stored at -80° C until analysis. The amount of prolactin in conditioned media was determined with an enzyme amplified sensitivity immunoassay (Catalog#KAQ1441; Biosource International, Hopkinton, MA). The amount of IL-6 conditioned media was determined with the Human IL-6 Quantikine ELISA Kit (catalog #D6050; R&D Systems, Minneapolis, MN). Data shown are means (*columns*) ± S.D. (*bars*) from three independent experiments performed in duplicate.

### Statistical analysis

For all experiments, at least three independent experiments were performed with *n*≥3 replicate samples per experiment. No statistical method was used to predetermine sample size. Investigators were not blinded to data allocation and experiments were not randomized. Data are presented as mean ± S.D. Comparisons of means of ≥3 groups were performed by one-way analysis of variance (ANOVA) and Dunnett’s t-test for multiple comparisons using XLSTAT 2010 (Addinsoft, Long Island, NY). In all studies, *P*-values <0.05 and <0.005 were considered to be statistically significant (denoted as * and **, respectively). All statistical tests were two-sided.

## Supplementary Material

Supplementary Figure 1
