# Supplementary material for: Progesterone receptor isoform-dependent cross-talk between prolactin and fatty acid synthase in breast cancer
Source: Aging (Albany NY). 2020 Dec 10;12(24):24671–92. doi: 10.18632/aging.202289 (PMC7803566; doi:10.18632/aging.202289)
Supplement: Supplementary Figure 1 [file aging-12-202289-s001.pdf]

SUPPLEMENTARY FIGURE

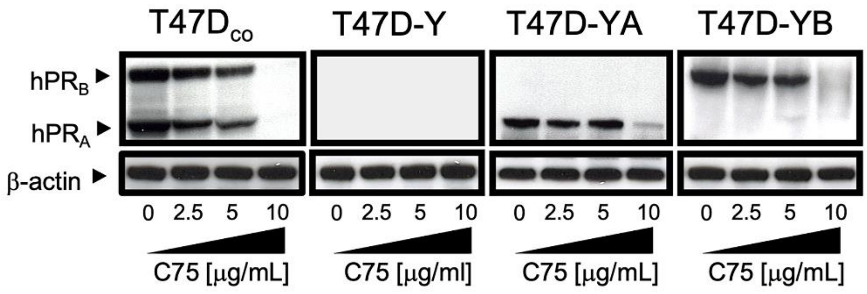

Supplementary Figure 1. Effects of FASN inhibition on expression of progesterone receptor isoforms. Immunoblotting of PR-A and PR-B proteins in $T47D_{co}$, T47D-Y, T47D-YA and T47D-YB breast cancer cell lines cultured in the absence or presence of graded concentrations of C75. β-actin was used to control for protein loading and transfer.
